# Diagnosing risk factors alongside mass drug administration using serial diagnostic tests—which test first?

**DOI:** 10.1093/trstmh/try062

**Published:** 2018-07-18

**Authors:** Louise Dyson, T Déirdre Hollingsworth

**Affiliations:** 1 Mathematics Institute, University of Warwick, Zeeman Building, Coventry, UK; 2 School of Life Sciences, University of Warwick, Gibbet Hill Campus, Coventry, UK; 3 Big Data Institute, Li Ka Shing Centre for Health Information and Discovery, University of Oxford, Old Road Campus, Oxford, UK

**Keywords:** coverage, diagnostics, mass screen and treat (MSAT), population-level impact, sensitivity, specificity

## Abstract

**Background:**

When tests are used in series to determine individual risk factors and infection status in a mass drug administration (MDA), the diagnostics, test order and subsequent treatment decisions (the testing algorithm) affect population-level treatment coverage and cost, but there is no existing framework for evaluating which algorithm optimizes any given outcome.

**Methods:**

We present a mathematical tool (with spreadsheet implementation) to analyse the effect of test ordering, illustrated using treatment for onchocerciasis in an area where high-burden *Loa loa* co-infections present a known risk factor.

**Results:**

The prevalence of the infection and risk factor have a non-linear impact on the optimal ordering of tests. Testing for the MDA infection first always leaves more infected people untreated but fewer people with the risk factor being misclassified. The cost of the treatment given to infected individuals with the risk factor does not affect which algorithm is more cost effective.

**Conclusions:**

For a given test and treat algorithm and its costs, the correct strategy depends on the expected prevalence. In most cases, when the apparent prevalence of the target infection is greater than the apparent prevalence of the risk factor, it is cheaper to do the risk factor test first, and vice versa.

## Introduction

As we approach disease elimination, it becomes increasingly important to be able to enact interventions everywhere the disease is endemic. This may require us to revisit communities, or individuals, in which treatment is complicated by the presence of risk factors that affect the treatment required, particularly for mass drug administration (MDA), a mainstay of neglected tropical disease (NTD) control.^1^ The presence of risk factors can prevent a drug from being safely administered to the whole population, reducing the coverage of the campaign both through a reduction in population that can be safely treated and through reduced local support due to adverse reactions to the treatment. This undermines the MDA campaign, which requires high coverage in order to be effective. Although this problem is particularly relevant for NTDs, it is also seen in other diseases, and indeed the infrequent use of MDA outside of NTDs is largely due to the lack of treatments with the right safety profile.

The combination of risk factors and multiple treatment regimens and diagnostic tests means that the design of the testing algorithm needs to be carefully considered. For example, there has recently been renewed interest in the possibility of MDA campaigns for malaria,^2,3^ which could be MDA or mass screen and treat (MSAT).^2,4^ For an MSAT campaign where pregnancy is a risk factor,^5^ there might be a question of whether to offer women of child-bearing age a pregnancy test or a malaria test first. There are many ethical considerations in this scenario, but there are also practical, cost and efficacy considerations that would affect the choice of test and the treatment algorithm.^6^

Another example of screening forming part of an MDA is for onchocerciasis in areas that are co-endemic with *Loa loa* filariasis. Where these diseases are co-endemic, the usual MDA treatment (ivermectin) has a high risk of severe adverse events (SAEs) if the individual has a high *Loa loa* microfilarial (mf) load.^7^ Under the current World Health Organization guidelines, areas with hypo-endemic onchocerciasis prevalence where *Loa loa* is known to be present have been left untreated.^8,9^ The question of how to eliminate onchocerciasis by delivering safe treatment in these co-endemic areas has recently been much debated.^10–12^ The development of the LoaScope,^13^ a mobile telephone–based video microscope, has made testing for *Loa loa* in the field more practical, leading to the ‘test-and-not-treat strategy’, where individuals with high *Loa loa* loads are identified through individual-level testing and not treated for onchocerciasis. This has recently been shown to be implementable in a population of more than 20 000 people.^8^

**Figure 1.**
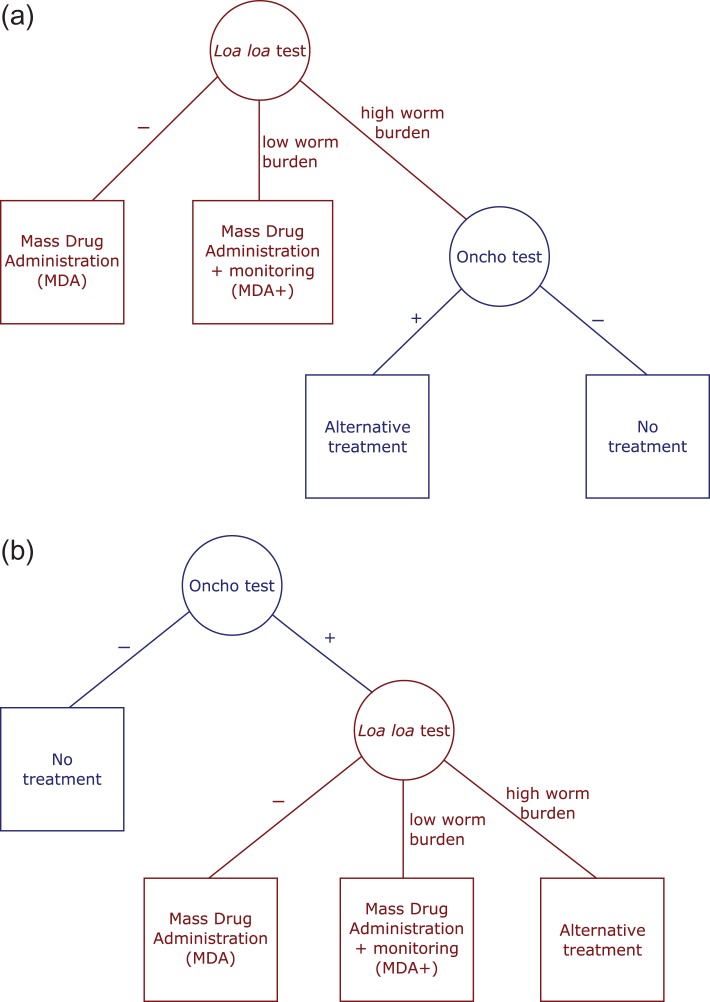
Example algorithms for test and treat for onchocerciasis and *Loa loa*. (a) *Loa loa* first. (b) Onchocerciasis first. The proportion of the population going down each part of the test-and-treat pathway depends on which test is administered first and the apparent prevalence of the MDA infection and the risk factor. Total costs depend on the proportion of the population that are given each test and the proportions taking each treatment.

Prior to this demonstration of field implementation, there were concerns that an approach that required testing the whole population for *Loa loa* would be too time consuming for large-scale screening. In response, there was a proposal to possibly combine the *Loa loa* test with an onchocerciasis test and only use the *Loa loa* test for a small part of the population. For this example, the primary aim is to avoid SAEs by constraining the testing algorithm to ensure that everyone who is given the standard treatment for onchocerciasis has been tested for *Loa loa.* There are two possible testing algorithms (Figure 1):

1. Screen the population using a risk factor test and then treat everyone who is negative, increasing MDA treatment coverage but, depending on the relative costs of the person-time running the tests, increasing costs.
2. Screen with the MDA infection test and only use a risk factor test on those who are positive for the infection (in our example, treatment coverage is constrained by the low sensitivity of the onchocerciasis rapid test at low prevalence).

It is not immediately obvious which of these algorithms is ‘best’ in terms of efficacy and cost. This article seeks to address that question by developing a general framework applying it to this onchocerciasis/*Loa loa* example.

It is important to note that since our research was undertaken a different solution is close to being implemented for onchocerciasis, in which each implementation unit is mapped to determine whether both onchocerciasis and *Loa loa* are present and, if so, each individual gives a blood sample that is tested for both diseases before treatment, increasing the sensitivity of the onchocerciasis test.

However, to our knowledge, the literature still lacks a framework for making a choice between serial testing and treatment algorithms on the basis of population-level impact. We note that a related question has been more widely studied in which multiple tests exist for the same disease, with different costs, specificities and sensitivities (e.g., for human immunodeficiency virus [HIV]).^14^ Again, the question of the ordering of these tests, which has been examined to determine the optimal ordering for diagnosis and cost, could be addressed using the tool outlined here. Our framework is relevant to any scenario in which a disease is present in a population that also displays some risk factor that increases the risk of SAEs when individuals are treated for a disease. It is important to note that this requires a test for the risk factor for which the sensitivity and specificity have been reliably determined under the circumstances in which the tool will be used.

The three outcomes we wish to balance are the number of people at risk of SAEs, the number of people infected with the disease that do not receive treatment and the total cost of the scheme, including the costs of tests and treatments. Factors that affect these outcomes include the sensitivities and specificities of the tests, the prevalence of the disease and the risk factor and the costs of the tests and treatments. We describe the methodology used in the tool to calculate the outcomes and the general patterns that emerge. We have produced a spreadsheet tool that allows the user to specify these different factors and explore their effects on the described outcomes. We illustrate how the tool can be used to assess the optimal order of test and treatment decisions for onchocerciasis scenarios, but the same methods may be easily applied to compare proposed treatment algorithms for any disease with a co-endemic risk factor.

## Methods

We consider a population of individuals, divided into six groups according to their onchocerciasis infection status (uninfected vs infected) and three levels of possible *Loa loa* infection (uninfected vs low mf load vs high mf load). How best to specify the joint distribution of the two diseases (or MDA infection and risk factor) depends on the set of initial assumptions. If the two diseases are presumed to be independent, then the joint distribution is just the product of the proportion of individuals with each disease. In the case of *Loa loa*, the proportion of the population that have no, low or high burdens of *Loa loa* can be specified by assuming a negative binomial mf distribution and a threshold above which the burden is considered to be ‘high’. Alternatively we can consider that the two infections may be correlated (as seen by Kelly-Hope et al.^15^), whereby having one disease may make infection with the second more likely. While this last specification is likely to be the most accurate, it requires additional data to inform the proportion of the population in each group and only affects our analysis of how the relative costs of the two schemes relate to the prevalence of the two diseases.

Note that our analysis of the risk of SAEs and the number of people with untreated onchocerciasis depends on both the true infection status of individuals as well as their test results. In contrast, our analysis of the relative costs of the two schemes relies only on the outcome of tests and not on the true underlying status.

Each of the six groups is then put through the two algorithms: testing for *Loa loa* first vs testing for onchocerciasis first (Figure 1). At each stage we take into account the specificity and sensitivity of that test. For example, if we take a group of size *N* that is uninfected with onchocerciasis, then, on average, (1−*sp*)*N* will test positive for onchocerciasis (false positives), where *sp* is the specificity of the test. Conversely, in a group of size *N* that is infected with onchocerciasis (1−*sens*)*N* will test negative (false negatives), where *sens* is the sensitivity. This procedure gives, for each of the six groups, the proportion that receives each of the different treatments, and thus the number at risk of SAEs and the number that are infected with onchocerciasis but are not treated. In addition, we calculate the total cost of the algorithm using the apparent prevalences of the various subgroups.

Spreadsheets implementing this analysis assuming either independence of the two diseases (TestnTreat_prevalences_independent.xlsx) or requiring co-infection information (TestnTreat_prevalences_independent.xlsx) are provided as supplementary files.

### Assumptions

Since the difference between negative and low burdens of *Loa loa* has little impact on treatment decision in our analysis, we will simplify the analysis by assuming that the *Loa loa* test always correctly identifies the presence or absence of *Loa loa*. Therefore in this article, the specificity and sensitivity of the *Loa loa* test refer only to whether the individual has a high or low mf burden. It would be straightforward to compute a similar analysis including uncertainty between negative and low *Loa loa* tests, and our results would still hold.

## Results

We now present an analysis of the two algorithms in Figure 1 using the groups found in Table 1.

**Table 1.** Tests and treatments employed for each algorithm (see Figure 1) stratified by how individuals test for the two diseases. Differences between the two schemes are highlighted in bold

(a) *Loa loa* test first (algorithm A)				
	*Loa loa* test result			
		−	Low	High
Onchocerciasis test result	−	***Loa loa*****test** **MDA**	***Loa loa*****test** **MDA+**	***Loa loa*****test** Onchocerciasis test
	+	*Loa loa* test MDA	*Loa loa* test MDA+	*Loa loa* test Onchocerciasis test Alternative treatment
(b) Onchocerciasis test first (algorithm B)				
	*Loa loa* test result			
		−	Low	High
Onchocerciasis test result	−	**Onchocerciasis test**	**Onchocerciasis test**	Onchocerciasis test
	+	**Onchocerciasis test**	**Onchocerciasis test**	Onchocerciasis test
		*Loa loa* test MDA	*Loa loa* test MDA+	*Loa loa* test Alternative treatment

### Testing for MDA infection first always results in more untreated infected people

In general, using the MDA infection or, in the example, onchocerciasis test first results in a larger proportion of people who are infected with onchocerciasis and are not given a treatment. This can be seen by calculating the number of infected people that are untreated under each scheme. If $N_{O^{+}}$ is the number of people who have onchocerciasis and we test for onchocerciasis first, then the number of untreated infected people is simply those who incorrectly tested negative for onchocerciasis, that is,

(1) $$\left(1\;-\;O_{\mathrm{sens}}\right)N_{O^{+}},$$

where O_sens_ is the sensitivity of the onchocerciasis test. Conversely, if we test for *Loa loa* first, then some people who would incorrectly test negative for onchocerciasis instead first test negative or low for *Loa loa* and thus receive treatment anyway. More specifically,

(2) $$\left(1\;-\;O_{\mathrm{sens}}\right)\left(\left(1\;-\;L_{\mathrm{sp}}\right)N_{L^{L}{,O}^{+}}\;+\;{\;L}_{\mathrm{sens}}N_{L^{H}{,O}^{+}}\right)$$

is the number of people infected with onchocerciasis who are untreated, where L_sp_ and L_sens_ are the specificity and sensitivity of the *Loa loa* test, respectively, and $N_{L^{L},O^{+}}$ and $N_{L^{H},O^{+}}$ are the number of onchocerciasis-positive people who have low and high *Loa loa* burdens, respectively. Since $N_{L^{L},O^{+}}\;+\;N_{L^{H},O^{+}}\;\leq \;N_{O^{+}}$ and (1−L_sp),L_sens≤1, then

(3) $$\left(1\;-\;O_{\mathrm{sens}}\right)\left(\left(1\;-\;L_{\mathrm{sp}}\right)N_{L^{L}{,O}^{+}}\;+\;{\;L}_{\mathrm{sens}}N_{L^{H}{,O}^{+}}\right)\;\leq \;\left(1\;-\;O_{\mathrm{sens}}\right)N_{O^{+}}$$

and so testing for onchocerciasis first results in more untreated infected people than testing for *Loa loa* first. More generally, if one test can lead directly to individuals not being considered for treatment (and the other does not), then doing that test first will lead to more untreated individuals.

### Testing for MDA infection first always results in fewer people at risk of SAEs

For similar reasons, testing for onchocerciasis first also results in fewer people at risk of SAEs. In this case, people who have high *Loa loa* burdens and receive an MDA treatment are considered to be at risk of SAEs. Similar to the above section, there will be fewer people in this category when testing for onchocerciasis first, since some people who have high *Loa loa* burdens will (rightly or wrongly) test negative for onchocerciasis and thus receive no treatment. We may again calculate the number of people at risk of SAEs under each scheme. When the *Loa loa* test is given first, the number of people with a high *Loa loa* burden that (incorrectly) test low for *Loa loa* (and thus are at risk of SAEs) is given by

(4) $$\left(1\;-\;L_{\mathrm{sens}}\right)\left(N_{L^{H}{,O}^{+}}\;+\;N_{L^{H}{,O}^{-}}\right),$$

where $N_{L^{H},O^{-}}$ is the number of people with a high *Loa loa* burden without onchocerciasis. Conversely, if the onchocerciasis test is given first, then only those testing positive for onchocerciasis will be considered for treatment and of these, some will incorrectly test low for *Loa loa*. The following population,

(5) $$\left(1\;-\;L_{\mathrm{sens}}\right)\left(O_{\mathrm{sens}}N_{L^{H}{,O}^{+}}\;+\;\left(1\;-\;O_{\mathrm{spec}}\right)N_{L^{H}{,O}^{-}}\right),$$

will therefore be at risk of SAEs. Since O_sens_≤1 and (1−O_sp_)≤1, then

(6) $$\left(1\;-\;L_{\mathrm{sens}}\right)\left(N_{L^{H}{,O}^{+}}\;+\;N_{L^{H}{,O}^{-}}\right)\left(1\;-\;L_{\mathrm{sens}}\right)\left(O_{\mathrm{sens}}N_{L^{H}{,O}^{+}}\;+\;\left(1\;-\;O_{\mathrm{spec}}\right)N_{L^{H}{,O}^{-}}\right),$$

and so testing for onchocerciasis first always results in fewer people at risk of SAEs than testing for *Loa loa* first. In general, if one test leads directly to people not being treated, and there is a risk of SAEs, then doing that test first will result in fewer people at risk of SAEs.

### Costs of the two schemes

The tests and treatments that would be given under the two schemes are summarised in Table 1. The population is divided into six subpopulations based on their test results for the two diseases, then for each subpopulation we give the tests and treatments given for that subpopulation under the scheme. So, for example, if an individual tests positive for onchocerciasis (either because they have onchocerciasis or because they receive a false positive on the test) and demonstrates a low *Loa loa* burden on their test, then on the *Loa loa*–first scheme (algorithm A) they will receive the *Loa loa* test and, on the basis of that, will be given the MDA treatment and will then be monitored for side effects. On the onchocerciasis-first scheme (algorithm B) they will be given the onchocerciasis test and, on the basis of that, will be given the *Loa loa* test and, on the basis of those two results, will receive MDA treatment plus monitoring for side effects.

### The cost of the alternative treatment does not affect which scheme is more cost effective

To determine which scheme is more efficient we only need to consider the differences between the two schemes. In particular, the alternative treatment is only given to people who test positive for onchocerciasis and test high for *Loa loa*. Since this is the subgroup that we are required to identify, this group always receives both tests and the alternative treatment. Therefore the cost of the alternative treatment does not affect which scheme is less expensive.

### Which is cheaper?

The price of tests and treatment depends only on the outcomes of those tests and not the true disease status of the individuals tested. We will therefore define $N_{X}^{T}$ to be the number of people that test as *X*. For example, $N_{L^{h}O^{-}}^{T}$ is the number of people that, if they were tested, would present with a high *Loa loa* burden and would be negative for onchocerciasis, regardless of their actual disease status. The difference in cost between the two schemes can be seen by using the information in bold in Table 1. This is given by (*Loa loa* first—onchocerciasis first):

(7) $$\begin{array}{c} N_{L^{-}O^{-}}^{T}\left(\;\left[\mathrm{MDA}\right]\;+\;L_{\mathrm{test}}\;-\;{\;O}_{\mathrm{test}}\right)\;+\;N_{L^{L}O^{-}}^{T}\;\left({\left[\mathrm{MDA}\right]}^{+}\;+\;{\;L}_{\mathrm{test}}\;-\;O_{\mathrm{test}}\right)\\ \;+N_{L^{H}O^{-}}^{T}L_{\mathrm{test}}\;-\;N_{L^{-}O^{+}}^{T}O_{\mathrm{test}}\;-\;N_{L^{L}O^{+}}^{T}O_{\mathrm{test}}, \end{array},$$

where L_test_, O_test_, [MDA] and [MDA]^+^ are the costs of the *Loa loa* test, onchocerciasis test, MDA treatment and MDA treatment plus observation, respectively. Rearranging and using $N_{L^{-}O^{-}}^{T}\;+\;N_{L^{-}O^{+}}^{T}\;=\;N_{L^{-}}^{T}$, $N_{L^{L}O^{-}}^{T}\;+\;N_{L^{L}O^{+}}^{T}\;=\;N_{L^{L}}^{T}$ and $N_{L^{-}O^{-}}^{T}\;+\;N_{L^{L}O^{-}}^{T}\;+\;N_{L^{H}O^{-}}^{T}\;=\;N_{O^{-}}^{T}$, we obtain

(8) $$L_{\mathrm{test}}N_{O^{-}}^{T}\;-\;{\;O}_{\mathrm{test}}\left(N_{L^{-}}^{T}\;+\;N_{L^{L}}^{T}\right)\;+\;\left[\mathrm{MDA}\right]N_{L^{-}O^{-}}^{T}\;+\;{\left[\mathrm{MDA}\right]}^{+}N_{L^{L}O^{-}}^{T}.$$

We may see from this that the *Loa loa*–first scheme (algorithm A) is more likely to be cheaper at lower onchocerciasis prevalences and/or when the *Loa loa* test, the MDA treatment or the MDA treatment with observation are more expensive.

Up to this point we have made no assumptions about how the prevalence of the two infections affect each other. For example, it may be the case that individuals with onchocerciasis are also more likely to have high loads of *Loa loa*. To progress with the analysis from here, however, we will assume that the two infections (and testing positive for the infections) are independent of each other. That is, individuals infected with onchocerciasis are no more or less likely to have *Loa loa* infections. It is convenient at this point to use apparent prevalences ($n_{X}^{T}\;=\;N_{X}^{T}/N$) rather than populations ($N_{X}^{T}$), so that combinations of populations are easily calculated. For example, the fraction of the population that tests negative for *Loa loa* and positive for onchocerciasis is given by $n_{L^{-}O^{+}}^{T}\;=\;n_{L^{-}}^{T}n_{O^{+}}^{T}$, and equation (8) becomes

(9) $$L_{\mathrm{test}}n_{O^{-}}^{T}\;-\;O_{\mathrm{test}}\left(1\;-\;n_{L^{h}}^{T}\right)\;+\;\left[\mathrm{MDA}\right]\;\left(1\;-\;n_{L^{-}}^{T}n_{O^{+}}^{T}\right)\;+\;{\left[\mathrm{MDA}\right]}^{+}\left(1\;-\;n_{L^{L}}^{T}n_{O^{+}}^{T}\right),$$

using $n_{O^{-}}^{T}=1-n_{O^{+}}^{T}$ and $n_{L^{-}}^{T}\;+\;n_{L^{L}}^{T}+1-n_{L^{h}}^{T}$. Solving equation (9) to find when the two schemes are equal gives

(10) $$n_{O^{+}}^{T}\;=\;1–\frac{\left(1\;-\;n_{L^{h}}^{T}\right)O_{\mathrm{test}}}{\left[\mathrm{MDA}\right]\left(1\;-\;n_{L^{h}}^{T}\right)\;+\;L_{\mathrm{test}}\;+\;\left({\left[\mathrm{MDA}\right]}^{+}\;-\;\left[\mathrm{MDA}\right]\right)n_{L^{L}}^{T}}.$$

**Figure 2.**
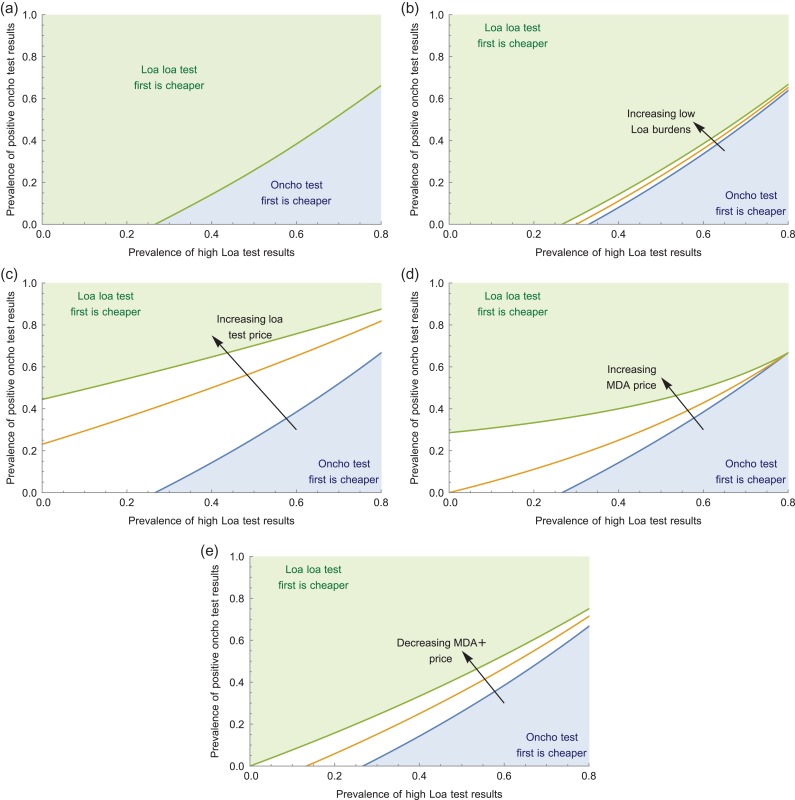
Investigating the effect of changing test and treatment prices on which scheme is cheaper. (a) The top dividing line shows where the two schemes cost an equal amount taking the parameter values: $\left[\mathrm{MDA}\right]=0.5,{\left[\mathrm{MDA}\right]}^{+}=1,O_{\mathrm{test}}=2,L_{\mathrm{test}}=1,n_{L^{L}}^{T}=0.2$. (b)–(e) We change different values in the way indicated by the arrow to go from the bottom dividing line to the top dividing line. Note that the prevalence of high *Loa loa* test results ($n_{L^{h}}^{T}$) is at most 0.8 since $n_{L^{-}}^{T}\;+\;n_{L^{L}}^{T}\;+\;n_{L^{h}}^{T}=1$ and we take $n_{L^{L}}^{T}\;=0.2$.

For $n_{O^{+}}^{T}$ greater than in equation (10), it is cheaper to do the *Loa loa* test first, whereas for lower onchocerciasis prevalences it is cheaper to do the onchocerciasis test first (algorithm B). It is intuitive that low onchocerciasis prevalences result in the onchocerciasis-first algorithm being cheaper, since people who test negative for onchocerciasis receive no further tests or treatment. Figure 2a gives an indication of the shape of these different regions. We note that $n_{L^{H}}^{T}$ is at most $1-n_{L^{L}}^{T}$, since we keep $n_{L^{L}}^{T}$ as a parameter and require that $n_{L^{H}}^{T}\;+\;n_{L^{L}}^{T}\;+\;n_{L^{-}}^{T}=1$.

Since the costs of the various tests and treatments may be variable depending on the situation, we explore the effect of changing these parameters on this plot. It is clear from equation (10) that the price of the onchocerciasis test merely scales with changes in the other parameters. In addition, increasing the apparent prevalence of low *Loa loa* burdens ($n_{L^{L}}^{T}$; Figure 2b), increasing the price of the *Loa loa* test ($L_{\mathrm{test}}$; Figure 2c) or decreasing the price of MDA treatment with observation ([MDA]^+^; Figure 2e) will have a similar effect. All of these changes will increase the number of positive onchocerciasis test results required before the *Loa loa*–first scheme (algorithm A) becomes cheaper. Increasing the price of MDA treatment, however, will lead to a more curved dividing line, so that the region in which the onchocerciasis-first scheme is cheaper becomes much larger (Figure 2d). This is due to the subpopulation with negative onchocerciasis and *Loa loa* results being untreated in the onchocerciasis-first scheme (algorithm B), while receiving MDA treatment under the *Loa loa*–first scheme. Since more people receive MDA treatments under the *Loa loa*–first scheme, this becomes more expensive as the cost of the MDA treatments increases.

## Conclusions

We have presented an analysis of two test-and-treat schemes: testing for the disease first or for the risk factor first. In our example scenario, the disease is onchocerciasis and the risk factor is co-infection with *Loa loa*. This illustrates that testing for the disease first (algorithm B) will always result in more untreated infected people, but it will also result in fewer people at risk of SAEs. We note that if the sensitivity of the risk factor test is 100% (as is given for *Loa loa* by D’Ambrosio et al.^13^), then there are no people at risk of SAEs from either scheme. In this case the scheme used will depend on the relative costs of the two possibilities. At low MDA infection prevalences the risk factor–first scheme is likely to be cheaper and, since it will result in fewer untreated infected people, would be the better choice (assuming 100% risk factor test sensitivity). At higher MDA infection prevalences, however, the MDA disease test–first scheme (algorithm B) may be cheaper and so the decision may rest on how much each additional treated person costs. For any particular circumstance this may be calculated as $(1-O_{\mathrm{sens}})(N_{O^{+}}-(1-L_{\mathrm{sp}})N_{L^{L},O^{+}}\;-\;L_{\mathrm{sens}}N_{L^{H},O^{+}})$ divided by equation (8). Note that to determine this it may be necessary to use the specificities and sensitivities of the diagnostics to convert between true underlying prevalences and the proportion of positive test results. We have provided a spreadsheet tool to assist with determining the relative costs and benefits of the schemes with given test and treatment costs and prevalences of the two diseases (see supplementary file).

It should be noted that we only consider here the risk of SAEs, the cost of the schemes and the proportion of infected people receiving treatment. The tool does not consider practicalities such as the need to take blood samples at a particular time of the day due to the diurnal periodicity of the *Loa loa* microfilaria. Nor does the tool consider specific diagnostic tools. To test for *Loa loa*, one could use the CellScope Loa test, which has been shown to have 94% specificity and 100% sensitivity^13^ and requires blood samples in the middle of the day.^16^ For onchocerciasis one could use the rapid format OV-16 antibody test,^17^ which has demonstrated sensitivities between 76.5% and 81.1% with 100% specificity. We note that the OV-16 test was developed as a tool for mapping the prevalence of onchocerciasis, not as a diagnostic test to determine when individuals receive treatment, as antibody tests cannot distinguish between past and current infections. Nonetheless, the tool presented here is not test dependent and can be used in any situation in which the accuracy and cost of the different tests, the disease and risk factor prevalences and the cost of treatments are known.

The situation considered here shares some similarities with the question of parallel vs serial tests that has been analysed for diseases such as HIV^14,18^ or canine Leishmaniasis.^19^ However, when considering parallel vs serial tests, we are usually looking at using multiple tests to improve the sensitivity and specificity of diagnosing a single disease. For example, one might consider using a test with a high sensitivity but low specificity first, then using a high specificity test on those that tested positive to reduce the number of false positives. Alternatively, one could use both tests in parallel and consider the individual to be positive if he/she test positive on either test, thus increasing the sensitivity at the expense of a lower specificity. Instead, we consider a case in which we are testing for two different diseases or risk factors in order to determine the correct treatment strategy.

The following results are generically true in the context of MDA with a risk factor. If one test can lead to untreated individuals (and the other does not), then doing that test first will lead to more (or equal) untreated individuals. If one test leads directly to people not being treated, and there is a risk of SAEs, then doing that test first will result in fewer (or equal) people at risk of SAEs. In addition, if the aim of both schemes is to identify a specific subpopulation in order to administer a given treatment (the ‘alternative treatment’ in our scenario), then the cost of that treatment does not affect which scheme is more expensive.

## Supplementary data

Supplementary data are available at Transactions online (http://trstmh.oxfordjournals.org/).

## Supplementary Material

Supplementary Data

Supplementary Data
